# 1-(1-Adamantylmeth­yl)-1*H*-benzimidazole

**DOI:** 10.1107/S1600536811041018

**Published:** 2011-10-12

**Authors:** Jarmila Černochová, Marek Nečas, Ivo Kuřitka, Robert Vícha

**Affiliations:** a Department of Chemistry, Faculty of Technology, Tomas Bata University in Zlin, Nám. T. G. Masaryka 275, Zlín,762 72, Czech Republic; b Department of Chemistry, Faculty of Science, Masaryk University, Kamenice 5, Brno-Bohunice, 625 00, Czech Republic; c Polymer Centre, Faculty of Technology, Tomas Bata University in Zlin, Nám. T. G. Masaryka 275, Zlín,762 72, Czech Republic, and, Centre of Polymer Systems, University Institute, Tomas Bata University in Zlin, Nad Ovčírnou 3685, Zlín, 760 01, Czech Republic

## Abstract

The asymmetric unit of the title compound, C_18_H_22_N_2_, contains two independent mol­ecules which differ slightly with respect to the torsion angles involving the atoms joining the adamantyl and benzimidazole groups. The bond angles in the adamantane cage vary within the range 108.27 (9)–110.55 (10)°. The benzimidazole ring system in both mol­ecules is essentially planar, the maximum deviations from the best planes being 0.0134 (15) and 0.0229 (14) Å. In the crystal, weak C—H⋯π inter­actions link the molecules.

## Related literature

For the synthesis, spectroscopic characterization and biological activity of the title compound, see: Hille *et al.* (2011 ▶). For background to C(*sp* ^2^)—H⋯π inter­actions, see: Takahashi *et al.* (2010 ▶). For two polymorphs of a related structure, see: Lei & Zhou (2009 ▶); Zhang *et al.* (2010 ▶).
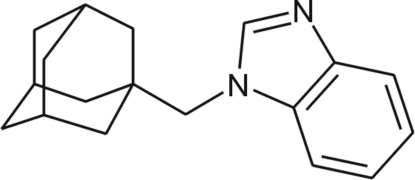


## Experimental

### 

#### Crystal data

C_18_H_22_N_2_
*M* *_r_* = 266.38
Monoclinic, 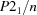

*a* = 22.0249 (9) Å
*b* = 6.4628 (1) Å
*c* = 22.2739 (8) Å
β = 118.694 (5)°
*V* = 2781.2 (2) Å^3^
*Z* = 8
Mo *K*α radiation
μ = 0.08 mm^−1^
*T* = 120 K
0.30 × 0.20 × 0.20 mm

#### Data collection

Oxford Diffraction Xcalibur Sapphire2 diffractometer
Absorption correction: multi-scan (*CrysAlis RED*; Oxford Diffraction, 2009 ▶) *T*_min_ = 0.928, *T*_max_ = 1.000
32353 measured reflections
4899 independent reflections
3314 reflections with *I* > 2σ(*I*)
*R*_int_ = 0.034

#### Refinement

*R*[*F* ^2^ > 2σ(*F* ^2^)] = 0.030
*wR*(*F* ^2^) = 0.064
*S* = 0.83
4899 reflections
361 parameters
H-atom parameters constrained
Δρ_max_ = 0.15 e Å^−3^
Δρ_min_ = −0.16 e Å^−3^

### 

Data collection: *CrysAlis CCD* (Oxford Diffraction, 2009 ▶); cell refinement: *CrysAlis RED* (Oxford Diffraction, 2009 ▶); data reduction: *CrysAlis RED*; program(s) used to solve structure: *SHELXS97* (Sheldrick, 2008 ▶); program(s) used to refine structure: *SHELXL97* (Sheldrick, 2008 ▶); molecular graphics: *ORTEP-3* (Farrugia, 1997 ▶) and *Mercury* (Macrae *et al.*, 2008 ▶); software used to prepare material for publication: *SHELXL97*.

## Supplementary Material

Crystal structure: contains datablock(s) global, I. DOI: 10.1107/S1600536811041018/lh5341sup1.cif

Structure factors: contains datablock(s) I. DOI: 10.1107/S1600536811041018/lh5341Isup2.hkl

Supplementary material file. DOI: 10.1107/S1600536811041018/lh5341Isup3.cml

Additional supplementary materials:  crystallographic information; 3D view; checkCIF report

## Figures and Tables

**Table 1:** Hydrogen-bond geometry (Å, °) *Cg*1 is the centroid of the C2–C7 ring.

*D*—H⋯*A*	*D*—H	H⋯*A*	*D*⋯*A*	*D*—H⋯*A*
C1—H1⋯*Cg*1^i^	0.95	3.07	3.9197 (16)	150

Symmetry code: (i) 
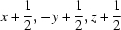
.

**Fig. 1.**
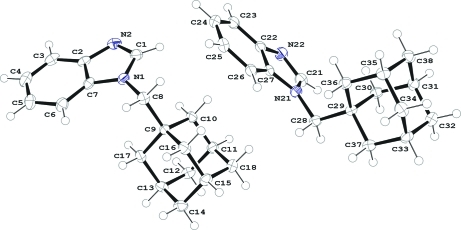
The asymmetric unit with 50% probability ellipsoids for non hydrogen atoms. H-atoms are shown as spheres at arbitrary radii.

**Fig. 2.**
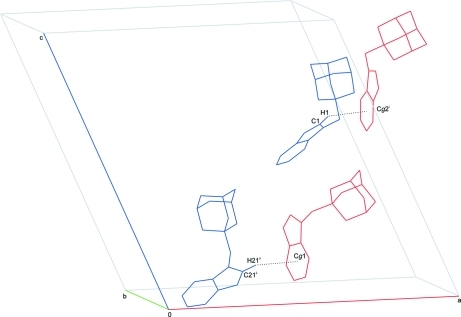
Two pairs of molecules linked via weak C—H···π interactions (dotted lines) are colored by symmetry equivalence. H-atoms are omitted except for those participating in H-bonds. Cg1 is centre of gravity of C2–C7, Symmetry codes: (i) x + 1/2, -y + 1/2, z + 1/2; (ii) x - 1/2, -y + 1/2, y - 1/2.

## supplementary crystallographic information

### Comment

Title compound has been prepared as a suitable building block for benzimidazolium-based carbene ligands synthesis and recently, the biological activity related to treatment of cortisole-dependent diseases has been studied (Hille *et al.*, 2011). Two polymorphs of a related structure have already been published (Lei & Zhou, 2009; Zhang *et al.*, 2010).

Both crystallographically independent molecules in the asymmetric unit (Fig. 1) contain essentially planar 1*H*-benzo[*d*]imidazole heterocycle with a maximum deviations from the best plane being 0.0134 (15) Å for C2 and 0.0229 (14) Å for C21, respectively. The torsion angles C7—N1—C8—C9 and N1—C8—C9—C16 describing the mutual orientation of benzimidazole and adamantane groups are 95.31 (15)° and -179.38 (10)°, respectively. The corresponding angles in the other molecule are -92.89 (15)° and -177.52 (10)°, respectively. The crystal packing is stabilized *via* weak C—H···π interactions (Fig. 2, Table 1).

### Experimental

Benzimidazole (0.40 g, 3.39 mmol) was dissolved in 40 cm^3^ of dry DMF and sodium hydride (0.2 g, 8.46 mmol) was added portionwise at room temperature. Into this mixture, 1-adamantylbromomethane (1.16 g, 5.09 mmol) was added and the mixture was stirred under argon for 5 days at 373 K. The reaction mixture was poured onto 100 g of crushed ice, extracted with 4 × 25 cm^3^ of dichloromethane and the collected organic portions were washed several times with distilled water, brine and dried over Na_2_SO_4_. The solvent was distilled off under reduced pressure and residual DMF was removed *via* azeotropic distillation with trichloromethane. The crude material was purified by crystallization (petroleum ether:ethyl acetate, 1:1, v:v) to yield 850 mg (94%) of colorless powder with mp=483–488 K. The crystal used for data collection was grown by spontaneous evaporation of a trichloromethane:methanol solution of the title compound at room temperature.

### Refinement

All carbon bound H atoms were placed at calculated positions and were refined as riding with their *U*_iso_ set to 1.2*U*_eq_ of the respective carrier atoms.

### Crystal data

**Table:**

C_18_H_22_N_2_	*F*(000) = 1152
*M**_r_* = 266.38	*D*_x_ = 1.272 Mg m^−^^3^
Monoclinic, *P*2_1_/*n*	Melting point: 486 K
Hall symbol: -P 2yn	Mo *K*α radiation, λ = 0.71073 Å
*a* = 22.0249 (9) Å	Cell parameters from 9351 reflections
*b* = 6.4628 (1) Å	θ = 2.8–27.3°
*c* = 22.2739 (8) Å	µ = 0.08 mm^−^^1^
β = 118.694 (5)°	*T* = 120 K
*V* = 2781.2 (2) Å^3^	Block, colourless
*Z* = 8	0.30 × 0.20 × 0.20 mm

### Data collection

**Table:**

Oxford Diffraction Xcalibur Sapphire2 diffractometer	4899 independent reflections
Radiation source: fine-focus sealed tube	3314 reflections with *I* > 2σ(*I*)
graphite	*R*_int_ = 0.034
Detector resolution: 8.4353 pixels mm^-1^	θ_max_ = 25.0°, θ_min_ = 3.2°
ω scans	*h* = −26→17
Absorption correction: multi-scan (*CrysAlis RED*; Oxford Diffraction, 2009)	*k* = −7→7
*T*_min_ = 0.928, *T*_max_ = 1.000	*l* = −26→26
32353 measured reflections	

### Refinement

**Table:**

Refinement on *F*^2^	Primary atom site location: structure-invariant direct methods
Least-squares matrix: full	Secondary atom site location: difference Fourier map
*R*[*F*^2^ > 2σ(*F*^2^)] = 0.030	Hydrogen site location: inferred from neighbouring sites
*wR*(*F*^2^) = 0.064	H-atom parameters constrained
*S* = 0.83	*w* = 1/[σ^2^(*F*_o_^2^) + (0.0354*P*)^2^] where *P* = (*F*_o_^2^ + 2*F*_c_^2^)/3
4899 reflections	(Δ/σ)_max_ < 0.001
361 parameters	Δρ_max_ = 0.15 e Å^−^^3^
0 restraints	Δρ_min_ = −0.16 e Å^−^^3^

### Special details

**Table:**

Geometry. All e.s.d.'s (except the e.s.d. in the dihedral angle between two l.s. planes) are estimated using the full covariance matrix. The cell e.s.d.'s are taken into account individually in the estimation of e.s.d.'s in distances, angles and torsion angles; correlations between e.s.d.'s in cell parameters are only used when they are defined by crystal symmetry. An approximate (isotropic) treatment of cell e.s.d.'s is used for estimating e.s.d.'s involving l.s. planes.
Refinement. Refinement of *F*^2^ against ALL reflections. The weighted *R*-factor *wR* and goodness of fit *S* are based on *F*^2^, conventional *R*-factors *R* are based on *F*, with *F* set to zero for negative *F*^2^. The threshold expression of *F*^2^ > 2σ(*F*^2^) is used only for calculating *R*-factors(gt) *etc*. and is not relevant to the choice of reflections for refinement. *R*-factors based on *F*^2^ are statistically about twice as large as those based on *F*, and *R*- factors based on ALL data will be even larger.

### Fractional atomic coordinates and isotropic or equivalent isotropic displacement parameters (Å^2^)

**Table:**

	*x*	*y*	*z*	*U*_iso_*/*U*_eq_	
N1	0.58857 (5)	0.07364 (14)	0.28113 (5)	0.0206 (2)	
N2	0.55730 (5)	0.39787 (15)	0.24104 (5)	0.0258 (3)	
C1	0.58788 (6)	0.27873 (18)	0.29503 (6)	0.0238 (3)	
H1	0.6080	0.3312	0.3404	0.029*	
C2	0.53553 (6)	0.26067 (18)	0.18631 (6)	0.0212 (3)	
C3	0.49849 (6)	0.2979 (2)	0.11594 (6)	0.0272 (3)	
H3	0.4847	0.4340	0.0986	0.033*	
C4	0.48257 (7)	0.1313 (2)	0.07262 (6)	0.0297 (3)	
H4	0.4574	0.1531	0.0246	0.036*	
C5	0.50261 (6)	−0.0698 (2)	0.09772 (6)	0.0290 (3)	
H5	0.4909	−0.1811	0.0662	0.035*	
C6	0.53897 (6)	−0.11098 (19)	0.16698 (6)	0.0249 (3)	
H6	0.5526	−0.2474	0.1840	0.030*	
C7	0.55451 (6)	0.05820 (18)	0.21038 (6)	0.0201 (3)	
C8	0.62286 (6)	−0.09207 (18)	0.33114 (6)	0.0221 (3)	
H8A	0.6009	−0.2255	0.3100	0.026*	
H8B	0.6150	−0.0687	0.3708	0.026*	
C9	0.70077 (6)	−0.10925 (17)	0.35702 (5)	0.0170 (3)	
C10	0.73908 (6)	0.08829 (17)	0.39375 (6)	0.0204 (3)	
H10A	0.7223	0.2061	0.3614	0.025*	
H10B	0.7293	0.1191	0.4318	0.025*	
C11	0.81690 (6)	0.06242 (18)	0.42177 (6)	0.0227 (3)	
H11	0.8412	0.1921	0.4458	0.027*	
C12	0.83131 (7)	0.01928 (18)	0.36233 (6)	0.0248 (3)	
H12A	0.8817	0.0039	0.3798	0.030*	
H12B	0.8148	0.1368	0.3298	0.030*	
C13	0.79412 (6)	−0.17918 (18)	0.32558 (6)	0.0222 (3)	
H13	0.8035	−0.2068	0.2866	0.027*	
C14	0.82057 (7)	−0.36073 (18)	0.37585 (6)	0.0255 (3)	
H14A	0.8709	−0.3786	0.3934	0.031*	
H14B	0.7970	−0.4899	0.3522	0.031*	
C15	0.80601 (7)	−0.31763 (18)	0.43533 (6)	0.0239 (3)	
H15	0.8231	−0.4360	0.4683	0.029*	
C16	0.72824 (7)	−0.29034 (18)	0.40795 (6)	0.0229 (3)	
H16A	0.7189	−0.2635	0.4465	0.027*	
H16B	0.7039	−0.4192	0.3849	0.027*	
C17	0.71637 (6)	−0.15487 (18)	0.29825 (6)	0.0210 (3)	
H17A	0.6924	−0.2835	0.2746	0.025*	
H17B	0.6988	−0.0403	0.2647	0.025*	
C18	0.84322 (7)	−0.11922 (18)	0.47179 (6)	0.0276 (3)	
H18A	0.8347	−0.0918	0.5108	0.033*	
H18B	0.8937	−0.1356	0.4899	0.033*	
N21	0.81236 (5)	0.11037 (14)	0.61146 (5)	0.0206 (2)	
N22	0.77666 (6)	0.43222 (15)	0.57082 (5)	0.0266 (3)	
C21	0.82934 (7)	0.31252 (19)	0.60867 (6)	0.0247 (3)	
H21	0.8756	0.3621	0.6323	0.030*	
C22	0.71997 (7)	0.29919 (18)	0.54557 (6)	0.0221 (3)	
C23	0.65052 (7)	0.3378 (2)	0.50043 (6)	0.0276 (3)	
H23	0.6351	0.4728	0.4828	0.033*	
C24	0.60493 (7)	0.1751 (2)	0.48206 (6)	0.0297 (3)	
H24	0.5572	0.1988	0.4516	0.036*	
C25	0.62717 (7)	−0.0254 (2)	0.50718 (6)	0.0279 (3)	
H25	0.5942	−0.1344	0.4931	0.033*	
C26	0.69569 (7)	−0.06793 (19)	0.55175 (6)	0.0231 (3)	
H26	0.7109	−0.2035	0.5689	0.028*	
C27	0.74142 (7)	0.09792 (18)	0.57037 (6)	0.0202 (3)	
C28	0.86030 (6)	−0.05658 (18)	0.65027 (6)	0.0211 (3)	
H28A	0.9047	−0.0329	0.6503	0.025*	
H28B	0.8411	−0.1891	0.6264	0.025*	
C29	0.87474 (6)	−0.07727 (17)	0.72453 (6)	0.0170 (3)	
C30	0.90524 (6)	0.12265 (17)	0.76505 (6)	0.0196 (3)	
H30A	0.9475	0.1611	0.7625	0.023*	
H30B	0.8713	0.2365	0.7447	0.023*	
C31	0.92319 (6)	0.09273 (18)	0.83991 (6)	0.0214 (3)	
H31	0.9433	0.2237	0.8658	0.026*	
C32	0.97601 (6)	−0.08287 (18)	0.87178 (6)	0.0252 (3)	
H32A	1.0189	−0.0479	0.8700	0.030*	
H32B	0.9879	−0.1014	0.9203	0.030*	
C33	0.94509 (7)	−0.28301 (18)	0.83216 (6)	0.0227 (3)	
H33	0.9794	−0.3980	0.8528	0.027*	
C34	0.87901 (7)	−0.33678 (18)	0.83533 (6)	0.0254 (3)	
H34A	0.8592	−0.4672	0.8102	0.031*	
H34B	0.8900	−0.3571	0.8835	0.031*	
C35	0.82659 (6)	−0.16154 (18)	0.80349 (6)	0.0223 (3)	
H35	0.7834	−0.1967	0.8056	0.027*	
C36	0.80937 (6)	−0.13371 (18)	0.72869 (6)	0.0208 (3)	
H36A	0.7895	−0.2636	0.7032	0.025*	
H36B	0.7744	−0.0228	0.7074	0.025*	
C37	0.92803 (6)	−0.25205 (17)	0.75754 (6)	0.0210 (3)	
H37A	0.9092	−0.3821	0.7317	0.025*	
H37B	0.9708	−0.2171	0.7556	0.025*	
C38	0.85747 (7)	0.03925 (18)	0.84316 (6)	0.0239 (3)	
H38A	0.8235	0.1531	0.8230	0.029*	
H38B	0.8683	0.0217	0.8915	0.029*	

### Atomic displacement parameters (Å^2^)

**Table:**

	*U*^11^	*U*^22^	*U*^33^	*U*^12^	*U*^13^	*U*^23^
N1	0.0174 (6)	0.0193 (6)	0.0220 (5)	0.0012 (5)	0.0070 (5)	0.0012 (4)
N2	0.0240 (7)	0.0219 (6)	0.0326 (6)	0.0042 (5)	0.0145 (5)	0.0022 (5)
C1	0.0203 (8)	0.0231 (7)	0.0282 (7)	0.0005 (6)	0.0118 (6)	−0.0036 (6)
C2	0.0135 (8)	0.0227 (7)	0.0282 (7)	0.0014 (6)	0.0104 (6)	0.0035 (5)
C3	0.0192 (8)	0.0300 (7)	0.0336 (7)	0.0041 (6)	0.0136 (6)	0.0109 (6)
C4	0.0196 (8)	0.0416 (9)	0.0237 (7)	−0.0017 (7)	0.0070 (6)	0.0063 (6)
C5	0.0239 (9)	0.0330 (8)	0.0249 (7)	−0.0076 (7)	0.0077 (6)	−0.0033 (6)
C6	0.0218 (8)	0.0231 (7)	0.0263 (7)	−0.0038 (6)	0.0087 (6)	0.0005 (6)
C7	0.0124 (8)	0.0242 (7)	0.0221 (7)	−0.0016 (6)	0.0071 (6)	0.0025 (5)
C8	0.0247 (8)	0.0200 (7)	0.0218 (7)	−0.0003 (6)	0.0113 (6)	0.0023 (5)
C9	0.0177 (8)	0.0164 (6)	0.0163 (6)	0.0014 (5)	0.0076 (6)	0.0010 (5)
C10	0.0235 (8)	0.0178 (6)	0.0190 (6)	0.0025 (6)	0.0094 (6)	−0.0011 (5)
C11	0.0193 (8)	0.0190 (7)	0.0244 (7)	−0.0009 (6)	0.0063 (6)	−0.0052 (5)
C12	0.0216 (8)	0.0223 (7)	0.0315 (7)	0.0026 (6)	0.0134 (7)	0.0043 (6)
C13	0.0258 (9)	0.0226 (7)	0.0232 (6)	0.0017 (6)	0.0156 (6)	−0.0014 (5)
C14	0.0254 (8)	0.0194 (7)	0.0329 (7)	0.0030 (6)	0.0149 (7)	−0.0008 (6)
C15	0.0253 (9)	0.0221 (7)	0.0221 (7)	0.0077 (6)	0.0097 (6)	0.0075 (5)
C16	0.0303 (9)	0.0203 (7)	0.0207 (6)	0.0020 (6)	0.0143 (6)	0.0025 (5)
C17	0.0262 (8)	0.0186 (6)	0.0171 (6)	−0.0011 (6)	0.0095 (6)	−0.0013 (5)
C18	0.0235 (8)	0.0330 (8)	0.0199 (7)	0.0052 (6)	0.0053 (6)	−0.0007 (6)
N21	0.0234 (7)	0.0184 (6)	0.0190 (5)	0.0037 (5)	0.0094 (5)	0.0028 (4)
N22	0.0324 (7)	0.0223 (6)	0.0233 (6)	0.0043 (6)	0.0119 (5)	0.0039 (5)
C21	0.0309 (9)	0.0223 (7)	0.0222 (7)	−0.0010 (6)	0.0138 (6)	0.0012 (6)
C22	0.0292 (9)	0.0221 (7)	0.0160 (6)	0.0058 (6)	0.0116 (6)	0.0011 (5)
C23	0.0354 (9)	0.0260 (7)	0.0185 (7)	0.0111 (7)	0.0108 (7)	0.0020 (6)
C24	0.0256 (9)	0.0367 (8)	0.0196 (7)	0.0097 (7)	0.0051 (6)	−0.0019 (6)
C25	0.0279 (9)	0.0321 (8)	0.0200 (7)	−0.0007 (7)	0.0085 (7)	−0.0054 (6)
C26	0.0280 (9)	0.0216 (7)	0.0176 (6)	0.0041 (6)	0.0093 (6)	−0.0005 (5)
C27	0.0224 (8)	0.0246 (7)	0.0131 (6)	0.0036 (6)	0.0083 (6)	−0.0016 (5)
C28	0.0205 (8)	0.0196 (6)	0.0229 (7)	0.0045 (6)	0.0102 (6)	0.0017 (5)
C29	0.0155 (7)	0.0163 (6)	0.0196 (6)	0.0009 (5)	0.0087 (6)	0.0015 (5)
C30	0.0160 (8)	0.0179 (6)	0.0258 (7)	0.0003 (5)	0.0108 (6)	0.0018 (5)
C31	0.0214 (8)	0.0181 (6)	0.0210 (6)	−0.0016 (6)	0.0074 (6)	−0.0019 (5)
C32	0.0221 (8)	0.0274 (7)	0.0221 (7)	0.0012 (6)	0.0073 (6)	0.0026 (6)
C33	0.0224 (8)	0.0193 (6)	0.0230 (7)	0.0060 (6)	0.0084 (6)	0.0053 (5)
C34	0.0348 (9)	0.0197 (7)	0.0235 (7)	−0.0012 (6)	0.0154 (6)	0.0027 (5)
C35	0.0206 (8)	0.0233 (7)	0.0264 (7)	−0.0019 (6)	0.0140 (6)	0.0014 (5)
C36	0.0183 (8)	0.0193 (6)	0.0238 (6)	−0.0002 (6)	0.0094 (6)	−0.0006 (5)
C37	0.0199 (8)	0.0184 (6)	0.0256 (7)	0.0014 (6)	0.0117 (6)	0.0003 (5)
C38	0.0284 (9)	0.0237 (7)	0.0220 (7)	0.0033 (6)	0.0141 (6)	0.0019 (5)

### Geometric parameters (Å, °)

**Table:**

N1—C1	1.3629 (14)	N21—C21	1.3685 (14)
N1—C7	1.3860 (14)	N21—C27	1.3829 (15)
N1—C8	1.4664 (13)	N21—C28	1.4661 (14)
N2—C1	1.3096 (14)	N22—C21	1.3100 (15)
N2—C2	1.3931 (15)	N22—C22	1.3929 (15)
C1—H1	0.9500	C21—H21	0.9500
C2—C3	1.3972 (16)	C22—C23	1.3915 (17)
C2—C7	1.3997 (16)	C22—C27	1.4035 (16)
C3—C4	1.3744 (17)	C23—C24	1.3738 (18)
C3—H3	0.9500	C23—H23	0.9500
C4—C5	1.4000 (17)	C24—C25	1.4029 (17)
C4—H4	0.9500	C24—H24	0.9500
C5—C6	1.3805 (16)	C25—C26	1.3784 (17)
C5—H5	0.9500	C25—H25	0.9500
C6—C7	1.3889 (16)	C26—C27	1.3908 (16)
C6—H6	0.9500	C26—H26	0.9500
C8—C9	1.5294 (16)	C28—C29	1.5329 (15)
C8—H8A	0.9900	C28—H28A	0.9900
C8—H8B	0.9900	C28—H28B	0.9900
C9—C10	1.5324 (15)	C29—C36	1.5306 (16)
C9—C17	1.5331 (15)	C29—C30	1.5340 (15)
C9—C16	1.5374 (15)	C29—C37	1.5380 (15)
C10—C11	1.5257 (16)	C30—C31	1.5299 (15)
C10—H10A	0.9900	C30—H30A	0.9900
C10—H10B	0.9900	C30—H30B	0.9900
C11—C12	1.5280 (16)	C31—C38	1.5234 (17)
C11—C18	1.5281 (16)	C31—C32	1.5337 (15)
C11—H11	1.0000	C31—H31	1.0000
C12—C13	1.5304 (16)	C32—C33	1.5292 (16)
C12—H12A	0.9900	C32—H32A	0.9900
C12—H12B	0.9900	C32—H32B	0.9900
C13—C17	1.5253 (16)	C33—C34	1.5305 (17)
C13—C14	1.5308 (16)	C33—C37	1.5310 (15)
C13—H13	1.0000	C33—H33	1.0000
C14—C15	1.5308 (16)	C34—C35	1.5273 (16)
C14—H14A	0.9900	C34—H34A	0.9900
C14—H14B	0.9900	C34—H34B	0.9900
C15—C16	1.5277 (16)	C35—C36	1.5314 (16)
C15—C18	1.5285 (16)	C35—C38	1.5331 (16)
C15—H15	1.0000	C35—H35	1.0000
C16—H16A	0.9900	C36—H36A	0.9900
C16—H16B	0.9900	C36—H36B	0.9900
C17—H17A	0.9900	C37—H37A	0.9900
C17—H17B	0.9900	C37—H37B	0.9900
C18—H18A	0.9900	C38—H38A	0.9900
C18—H18B	0.9900	C38—H38B	0.9900
			
C1—N1—C7	105.63 (10)	C21—N21—C27	105.78 (10)
C1—N1—C8	126.55 (10)	C21—N21—C28	126.46 (11)
C7—N1—C8	127.68 (10)	C27—N21—C28	127.76 (10)
C1—N2—C2	103.80 (10)	C21—N22—C22	103.92 (10)
N2—C1—N1	114.83 (11)	N22—C21—N21	114.59 (12)
N2—C1—H1	122.6	N22—C21—H21	122.7
N1—C1—H1	122.6	N21—C21—H21	122.7
N2—C2—C3	129.95 (11)	C23—C22—N22	129.96 (11)
N2—C2—C7	110.23 (10)	C23—C22—C27	119.75 (12)
C3—C2—C7	119.79 (11)	N22—C22—C27	110.24 (11)
C4—C3—C2	117.86 (12)	C24—C23—C22	118.07 (12)
C4—C3—H3	121.1	C24—C23—H23	121.0
C2—C3—H3	121.1	C22—C23—H23	121.0
C3—C4—C5	121.46 (12)	C23—C24—C25	121.53 (12)
C3—C4—H4	119.3	C23—C24—H24	119.2
C5—C4—H4	119.3	C25—C24—H24	119.2
C6—C5—C4	121.84 (12)	C26—C25—C24	121.56 (13)
C6—C5—H5	119.1	C26—C25—H25	119.2
C4—C5—H5	119.1	C24—C25—H25	119.2
C5—C6—C7	116.30 (12)	C25—C26—C27	116.53 (12)
C5—C6—H6	121.9	C25—C26—H26	121.7
C7—C6—H6	121.9	C27—C26—H26	121.7
N1—C7—C6	131.75 (11)	N21—C27—C26	131.96 (11)
N1—C7—C2	105.50 (10)	N21—C27—C22	105.45 (11)
C6—C7—C2	122.74 (11)	C26—C27—C22	122.55 (12)
N1—C8—C9	114.51 (9)	N21—C28—C29	114.71 (9)
N1—C8—H8A	108.6	N21—C28—H28A	108.6
C9—C8—H8A	108.6	C29—C28—H28A	108.6
N1—C8—H8B	108.6	N21—C28—H28B	108.6
C9—C8—H8B	108.6	C29—C28—H28B	108.6
H8A—C8—H8B	107.6	H28A—C28—H28B	107.6
C8—C9—C10	111.79 (9)	C36—C29—C28	111.85 (9)
C8—C9—C17	111.28 (9)	C36—C29—C30	108.90 (9)
C10—C9—C17	108.93 (9)	C28—C29—C30	111.52 (9)
C8—C9—C16	107.83 (9)	C36—C29—C37	108.67 (9)
C10—C9—C16	108.64 (9)	C28—C29—C37	107.17 (9)
C17—C9—C16	108.27 (9)	C30—C29—C37	108.62 (9)
C11—C10—C9	110.46 (9)	C31—C30—C29	110.23 (9)
C11—C10—H10A	109.6	C31—C30—H30A	109.6
C9—C10—H10A	109.6	C29—C30—H30A	109.6
C11—C10—H10B	109.6	C31—C30—H30B	109.6
C9—C10—H10B	109.6	C29—C30—H30B	109.6
H10A—C10—H10B	108.1	H30A—C30—H30B	108.1
C10—C11—C12	108.99 (10)	C38—C31—C30	109.04 (9)
C10—C11—C18	109.93 (10)	C38—C31—C32	109.70 (10)
C12—C11—C18	109.12 (10)	C30—C31—C32	109.85 (10)
C10—C11—H11	109.6	C38—C31—H31	109.4
C12—C11—H11	109.6	C30—C31—H31	109.4
C18—C11—H11	109.6	C32—C31—H31	109.4
C11—C12—C13	109.63 (10)	C33—C32—C31	109.28 (10)
C11—C12—H12A	109.7	C33—C32—H32A	109.8
C13—C12—H12A	109.7	C31—C32—H32A	109.8
C11—C12—H12B	109.7	C33—C32—H32B	109.8
C13—C12—H12B	109.7	C31—C32—H32B	109.8
H12A—C12—H12B	108.2	H32A—C32—H32B	108.3
C17—C13—C12	109.70 (10)	C32—C33—C34	109.59 (10)
C17—C13—C14	109.32 (10)	C32—C33—C37	108.97 (9)
C12—C13—C14	109.46 (10)	C34—C33—C37	109.67 (10)
C17—C13—H13	109.4	C32—C33—H33	109.5
C12—C13—H13	109.4	C34—C33—H33	109.5
C14—C13—H13	109.4	C37—C33—H33	109.5
C13—C14—C15	109.23 (10)	C35—C34—C33	109.50 (10)
C13—C14—H14A	109.8	C35—C34—H34A	109.8
C15—C14—H14A	109.8	C33—C34—H34A	109.8
C13—C14—H14B	109.8	C35—C34—H34B	109.8
C15—C14—H14B	109.8	C33—C34—H34B	109.8
H14A—C14—H14B	108.3	H34A—C34—H34B	108.2
C16—C15—C18	109.12 (10)	C34—C35—C36	109.23 (10)
C16—C15—C14	109.64 (10)	C34—C35—C38	109.44 (10)
C18—C15—C14	109.32 (10)	C36—C35—C38	109.71 (9)
C16—C15—H15	109.6	C34—C35—H35	109.5
C18—C15—H15	109.6	C36—C35—H35	109.5
C14—C15—H15	109.6	C38—C35—H35	109.5
C15—C16—C9	110.55 (10)	C29—C36—C35	110.19 (10)
C15—C16—H16A	109.5	C29—C36—H36A	109.6
C9—C16—H16A	109.5	C35—C36—H36A	109.6
C15—C16—H16B	109.5	C29—C36—H36B	109.6
C9—C16—H16B	109.5	C35—C36—H36B	109.6
H16A—C16—H16B	108.1	H36A—C36—H36B	108.1
C13—C17—C9	110.37 (9)	C33—C37—C29	110.37 (9)
C13—C17—H17A	109.6	C33—C37—H37A	109.6
C9—C17—H17A	109.6	C29—C37—H37A	109.6
C13—C17—H17B	109.6	C33—C37—H37B	109.6
C9—C17—H17B	109.6	C29—C37—H37B	109.6
H17A—C17—H17B	108.1	H37A—C37—H37B	108.1
C11—C18—C15	109.76 (10)	C31—C38—C35	109.47 (10)
C11—C18—H18A	109.7	C31—C38—H38A	109.8
C15—C18—H18A	109.7	C35—C38—H38A	109.8
C11—C18—H18B	109.7	C31—C38—H38B	109.8
C15—C18—H18B	109.7	C35—C38—H38B	109.8
H18A—C18—H18B	108.2	H38A—C38—H38B	108.2

### Hydrogen-bond geometry (Å, °)

**Table:**

*Cg*1 is the centroid of the C2–C7 ring.

**Table:**

*D*—H···*A*	*D*—H	H···*A*	*D*···*A*	*D*—H···*A*
C1—H1···Cg1^i^	0.95	3.07	3.9197 (16)	150.

Symmetry codes: (i) *x*+1/2, −*y*+1/2, *z*+1/2.
